# Prevalence and correlates of common mental disorders in people living with HIV in primary health care facilities in Ekurhuleni district

**DOI:** 10.4102/phcfm.v16i1.4568

**Published:** 2024-10-24

**Authors:** Aniekan Edet, Samuel Agbo, Afolake A. Amodu, Nwabisa N. Edet

**Affiliations:** 1 Department of Family Medicine and Primary Care, Faculty of Health Sciences, University of the Witwatersrand, Johannesburg, South Africa; 2 Limpopo Department of Health, Donald Fraser Hospital, Thohoyandou, South Africa; 3 Family Medicine and Primary Care Unit, Ekurhuleni Health District, Ekurhuleni, South Africa; 4 Department of Advanced Nursing Science, Faculty of Health Sciences, University of Venda, Thohoyandou, South Africa; 5 Gauteng Department of Health, Ekurhuleni Health District, Ekurhuleni, South Africa

**Keywords:** common mental disorders, depression, generalised anxiety disorder, substance-use disorder, PLHIV, CMDs, PHQ-9, GAD-7

## Abstract

**Background:**

There is paucity of data regarding the prevalence of common mental disorders (CMDs) in people living with HIV (PLHIV) in Ekurhuleni Health District (EHD), South Africa. Also, there is an association between CMDs and poor HIV treatment outcomes. Guidelines therefore recommend that healthcare practitioners screen for CMDs in PLHIV.

**Aim:**

To determine the prevalence and correlates of CMDs in PLHIV in primary health care facilities in Ekurhuleni district.

**Setting:**

Seven primary health facilities in Ekurhuleni district.

**Methods:**

A cross-sectional study was conducted in which data were collected from 403 randomly selected participants, using a questionnaire that incorporated the scores of the Patient Health Questionnaire (PHQ)-9, generalised anxiety disorder (GAD)-7 and substance use disorder (SUD) criteria of Diagnostic and Statistical Manual of Mental Disorders, Fifth Edition (DSM 5). The proportion screening positive for CMDs was calculated. ‘R’ statistical software was used for univariate and multivariate analysis, with a confidence interval (CI) of 95%.

**Results:**

Most participants (63%) were female and the mean age was 43 ± 11 years. Forty per cent of participants screened positive for CMDs, 16.6%, 15.1% and 24.1% screened positive for depression, GAD and SUD, respectively. Common mental disorders were associated with poor adherence and HIV non-suppression, while increasing age and being female were associated with reduced risk of CMDs. The risk of severe SUDs in males was 11 times compared to females. During assessment, clinicians screened only 16%, 14% and 40% of the cohort for depression, GAD and SUDs, respectively.

**Conclusion:**

The prevalence of CMDs remains high. Adherence to recommendations to screen for CMDs in PLHIV is low.

**Contribution:**

This study reveals a low CMD screening rate, estimates the prevalence of CMDs in PLHIV in Ekurhuleni district, and its impact.

## Introduction

Previous research has suggested that the incidence and prevalence of common mental disorders (CMDs) in people living with HIV (PLHIV) is higher than in the general population. One of these studies, conducted in South Africa in 2019, suggests a prevalence higher than five times that of the general population.^1,2,3^ This higher prevalence has been attributed to the initial feeling of uncertainty after the diagnosis of HIV, social stigma, social or mental isolation, and non-disclosure of HIV diagnosis. This may preclude PLHIV from receiving clinical and psychosocial support.^4^ Another study associated this higher prevalence to the direct effects of the human immunodeficiency virus (HIV) or the antiretroviral therapy (ART) drugs on the brain.^5,6^ However, the extent these associated factors contribute to the development of CMDs in PLHIV may have changed over time. For example, the periodic South African stigma index survey reported a significant reduction in HIV-related internalised stigma from 43.0% in 2014 to 23.4% in 2020.^7,8^ Similarly, there has been an increasing trend of HIV status disclosure to family members and partners.^9^ These variations in associated factors may have also triggered changes in the prevalence of these CMDs in PLHIV.

The more frequently occurring of these CMDs include depression, generalised anxiety disorder (GAD) and substance use disorders (SUDs).^10^ They have been associated with poor HIV treatment outcomes, such as decreased ART adherence, retention-in-care, HIV suppression, as well as, the overall quality of life.^11,12^ These CMDs may have negatively affected South Africa’s aspirations of achieving the third ‘90’ of the Joint United Nations Programme on HIV/AIDS (UNAIDS) 90-90-90 targets, which aimed to ensure that ‘90% of all persons receiving lifelong ART attain sustained viral suppression by the year 2020’.^12^ However, by March 2019, only 57.9% of persons who started lifelong ART were remaining on treatment, while 83.0% of those who had viral load tested achieved viral suppression, in Ekurhuleni district.^13^ Furthermore, only 63% – 88% of PLHIV were adherent to ART at 2 years after initiation, and 25.1% are lost-to-follow-up (LTF) at 5 years.^14,15^ These poor outcomes may be attributable in part to CMDs.^11,12^

The South African National Department of Health (SA NDoH) ART guidelines therefore recommend that healthcare practitioners screen PLHIV for CMDs prior to and after ART initiation.^16^ The degree of adherence to this recommendation in primary care has not been published. Some studies have further suggested that managing these CMDs in PLHIV may result in improved ART outcomes.^17^ This reinforces the importance of screening for CMDs in PLHIV. There is also a paucity of studies investigating the prevalence of CMDs in PLHIV in primary health care in Ekurhuleni district, and also, no study was found that estimated the proportion of PLHIV screened, by their treating clinician, for CMDs in the district, as recommended by the SA NDoH.^16^

The aim of this study is therefore to determine the prevalence of CMDs in public primary health care facilities in the eastern sub-district of Ekurhuleni district, South Africa, as well as, their associated factors. The study will also assess the proportion of PLHIV who were screened, by their treating clinician, for CMDs during their last two clinic visits.

## Research methods and design

### Study design

This was a cross-sectional, quantitative study using an investigator administered questionnaire.

### Study settings

The study site was the Eastern sub-district of Ekurhuleni. It is one of the three sub-districts of Ekurhuleni Health District (EHD). Excluding the mobile clinics, the Eastern sub-district had 30 primary health care clinics that were providing ART services to 85 509 adults as at 31 March 2020 utilising data from the district health information system (DHIS). Only one of the sub-districts was used for the study as all the sub-districts, as well as nationally, use the same guidelines in managing PLHIV.

Four of these healthcare facilities are open for 24 h, of which three are community health centres (CHCs) and one is a clinic. These four are also similar because they have full-time doctors and nurses, as well as allied health professionals, some of whom were available 1 day a week. These four facilities provide a comprehensive package of primary health care (PHC) services including preventive and/or promotive services such as immunisation, treatment services such as ART and rehabilitative services such as physiotherapy and optometry services. Mental health services are provided in these facilities by a psychologist, a mental health care nurse and psychiatry medical officers and registrars. The remaining 26 healthcare facilities were PHC day clinics, operating for 8 to 12 h daily, and were entirely nurse-led, with weekly support visits by doctors.

### Study population

The study population included PLHIV, who were 18 years and older, and a client at any public primary care clinic in the eastern sub-district of Ekurhuleni district, between 22 November 2021 and 25 March 2022. Persons who presented as a medical emergency, as well as, those who were not able to give consent, were excluded from the study.

### Sample size and sampling

Using an online sample size calculator, the sample size was 383 with a response distribution of 50%, a confidence interval (CI) of 95% and a 5% margin of error. The sample size was increased to 403 participants to allow for missing data.

A stratified random sampling technique was used to select facilities and participants. The healthcare facilities were grouped into two strata. The first stratum had 4 CHCs providing 24 h services, while the second stratum had 26 clinics that provided healthcare services for 8–12 h per day. Using proportionate sampling, one facility was chosen from stratum 1, while six facilities were chosen from stratum 2, by simple random sampling. Regarding participant selection, simple balloting was used to choose 109 participants from stratum 1 and 294 participants from stratum 2. The process of simple balloting involved placing folded papers that contained an equal number of ‘yes’ and ‘no’ in a box, before each participant was asked to pick one paper from the box. This was only after the participant had met the inclusion criteria and had given consent to be a part of the study. The process was continued until the sample size had been achieved.

### Study instrument

The researcher designed a questionnaire that included the scores of other previously validated tools, such as the Patient Health Questionnaire (PHQ)-9, the GAD-7 and the Diagnostic and Statistical Manual of Mental Disorders, Fifth Edition (DSM 5) criteria for SUD.^18,19^ The questionnaire was divided into five sections. Section 1 contained socio-demographic and clinical variables of participants. Section 2 assessed if participants had been screened for CMDs by the treating clinician based on recall and their clinic records during their last two visits. Section 3 contained the PHQ-9 score, while the fourth section contained the GAD-7 score. The PHQ-9 and GAD-7 have been previously validated in primary health care in South Africa and in local South African languages, including Isi-Zulu.^18,19^ Section 5 adopted a ‘Yes’ or ‘No’ response to each of the 11 DSM 5 criteria for SUD, which is the gold standard for diagnosis. A ‘yes’ response scored one and a ‘no’ response scored zero. A score of 2 or 3 indicates a mild SUD; a score of 4 or 5 indicates a moderate SUD, and a score of 6 or more criteria indicates a severe SUD.^20^ The SUD questions were administered for each substance used. Also, the copyrights for the PHQ-9 and the GAD-7 are owned by Pfizer Inc, and both questionnaires are publicly available and free to use without permission.^21,22^ Scores of 9 or more for PHQ-9, 8 or more for GAD-7, and the DSM 5 SUD criteria score of 2 or more were accepted as a positive screen for depression, GAD and SUD, respectively.

The instrument was reviewed by two family physicians for content validity. The inter-rater reliability was determined using the Kappa statistic which was found to be 0.83, which was considered optimal.^23^ A research assistant that was fluent in English language and Isi-Zulu assisted in translation of the content of the questionnaire to participants. Prior to commencement of data collection, the research assistant was trained in the translation of questionnaires, as well as, in research ethics to ensure confidentiality. The confidentiality of participants was also maintained by replacing their names with codes.

### Data collection

The seven healthcare facilities selected were visited 3 to 4 days a week from 22 November 2021 to 25 March 2022, recruiting 8–15 participants per day, requiring approximately 35 min per participant. The researcher approached all persons that met the inclusion criteria, and those who gave their written consent were included in the study. Each participant was separately taken to a private room where the questionnaire was administered. The questionnaire, which was paper-based, was completed using the verbal responses of participants and their clinic folders.

A clinic not selected for the study was used for a pilot study. This was done to determine feasibility of the study and to possibly improve on its design. Some logistical problems were discovered and were addressed. The pilot sample was not incorporated into the main research cohort. Participants who screened positive for any CMD were referred to a doctor in the facility for further assessment and management. One participant who was found to have suicidal ideation and intent while conducting the pilot study was immediately referred for management.

### Data analysis

The data collected were analysed using the ‘R’ statistical software and coded to a vector containing ‘0’ and ‘1’, representing the absence or presence of a variable, respectively. The number of categories per variable determined the number of vectors created. Data cleaning was carried out by reviewing each measured study variable; errors identified in the data cleaning process were corrected using participant information from the questionnaire. Variables were categorised and encoded based on previous studies and expert knowledge. The characteristics of the patients were presented using frequencies and percentages. The percentage of participants who had been screened for CMDs by their treating clinician was calculated. The percentages of those who screened positive for depression, GAD and SUDs during our study were also calculated.

Univariate logistic regression was conducted to assess for association between each measured variable and the response variable. Variables that were significant at a 20% significance level were included in the multivariable analysis. Previous studies have reported that the number of events per variable (EPV) is an important factor that could influence the degree of association between the variables and the outcome variables. A sample size with an EPV of 10 and more is usually recommended to avoid bias in the estimation.^24^ Therefore, the multivariable analysis was conducted with the backward elimination method of variable selection. In the final model, the selected variables are reported with their unadjusted odds ratio (OR) and adjusted odds ratio (aOR). A 95% CI was adopted, and a *p*-value of less than 0.05 was considered significant.

### Ethical considerations

Ethical approval to conduct the study was obtained from the University of the Witwatersrand Human Research Ethics Committee (Medical) (No. M210116). Ethics approval was also obtained from the Ekurhuleni Health District Ethics Committee (No. 11/02/2021-01).

Written informed consent form was obtained and signed by the participants who agreed to respond to the questionnaire and to participate in the study.

## Results

### Socio-demographic characteristics

Of the 403 participants, 63% were female (Table 1). The average age of the cohort was 43.0 ± 11.8 years, with a range of 18 to 87 years. Most (61%) of the questionnaires were administered in English language. Regarding employment status, 39% of the participants were employed on a full-time basis, 6% were receiving social grants or pension, while the rest of the participants had no regular source of income (Table 1).

**TABLE 1:** Socio-demographic characteristics of participants.

Characteristics	*n*	%
**Age (*N* = 403) (years)**		
18–30	62	15.0
31–40	113	28.0
41–50	121	30.0
> 50	107	27.0
**Sex (*N* = 403)**		
Male	151	37.0
Female	252	62.0
**Level of education (*N* = 395)**		
No matric	292	74.0
Matric or higher	103	26.0
**Relationship status (*N* = 402)**		
Married	70	17.0
Cohabitation	115	29.0
Single	216	54.0
**Presence of support system (*N* = 395)**		
Never	15	4.0
Sometimes	73	18.0
Always	307	78.0
**Experienced discrimination in the last 6 months (*N* = 393)**		
Yes	25	6.0
No	368	94.0

Importantly, although some questionnaires were not completely filled, the missing variables did not have any impact on the calculated sample size. The power of the study was therefore not affected by the incomplete variables. Another reason for the incomplete variables was that some participants had been on ART for less than 6 months, and as such did not yet have a viral load result. Analysis was therefore done using participants who had data on the variable of interest. Furthermore, some variables that were not significantly associated with any of the CMDs were not presented in some tables because of space restrictions. They can be made available upon request.

### Clinical characteristics

Viral suppression was defined at two thresholds; < 50 or < 200 copies/mL, to allow for ‘viral blips’ (Table 2). These thresholds were chosen because several HIV prevention studies suggest a ‘zero’ transmission when the viral load is less than 200 copies/mL. Using 50 copies/mL as the threshold, 70% of the participants had achieved viral suppression, while 82% of the participants achieved viral suppression when the threshold was increased to < 200 copies/mL. Most of the participants (91%) were on a Dolutegravir-based regimen. Only 67% of the cohort reported complete adherence to ART, while 23% reported experiencing chronic pain.

**TABLE 2:** Clinical characteristics of participants.

Characteristics	*n*	%
**Viral suppression (*N* = 364) (copies/mL)**		
< 50 copies/mL	254	70.0
> 50 copies/mL	110	27.0
**Viral suppression (*N* = 364) (copies/mL)**		
< 200 copies/mL	298	82.0
> 200 copies/mL	66	18.0
**Antiretroviral therapy type (*N* = 394)**		
EFV-based	107	27.0
DTG-based	253	64.0
PI-based	28	7.0
NVP-based	6	1.0
**Adherence in previous month (*N* = 395)**		
0 missed dose	263	67.0
1–2 missed dose	89	22.0
≥ 2 missed doses	43	11.0
Previous history of default (*N* = 394)	58	15.0
Disclosed HIV status (*N* = 395)	367	93.0
Other chronic diagnosis (*N* = 403)	145	36.0
Psychosocial stressors present (*N* = 403)	102	25.0
Chronic pain present (*N* = 400)	309	77.0
**Types of substance used (*N* = 403):**		
Alcohol	230	57.0
Tobacco	98	24.0
Cannabis	15	4.0
Opioid	2	0.5
No substance used	199	49.0

EFV, Efavirenz; DTG, Dolutegravir; PI, Protease Inhibitors; NVP, Nevirapine.

There were 48 participants who screened positive for alcohol-use disorder (AUD), 52% of whom had mild AUD, while 27% and 21% had moderate and severe AUD, respectively. Furthermore, 74 participants screened positive for tobacco-use disorder, 26% of which were mild, 48% were moderate and 26% were severe. Only eight participants screened positive for cannabis-use-disorder, while two screened positive for opioid-use-disorder. No participant reported using other substances.

### Prevalence of common mental disorders

**FIGURE 1:**
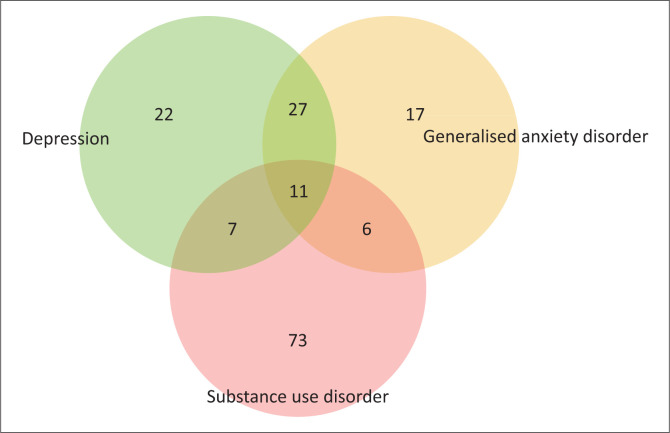
Frequency of comorbid common mental disorders among people living with HIV.

Sixty-seven (16.6%) of the participants screened positive for depression, 61 (15.1%) screened positive for GAD and 97 (24.1%) screened positive for SUDs. Of those that screened positive for SUDs, 48% had mild SUD, 26% had moderate SUD and 26% had severe SUD. While 163 (40%) persons in the cohort screened positive for CMDs, 112 (27.8%) screened positive for only one CMD, 40 (9.9%) screened positive for two CMDs and 11 (2.7%) of the participants had all three CMDs (Figure 1).

### Factors associated with common mental disorders among people living with HIV

Table 3 depicts the association between each study variable and CMD. The presence of chronic pain, level of education, experience of stigma or discrimination, availability of family support, relationship status, year of HIV diagnosis and the presence of psychosocial stressors were each not significantly associated in CMDs. However, screening positive for CMDs was significantly associated with having a viral load of >200 copies/mL (OR = 2.17; 95% CI: 1.26–3.72; *p* = 0.005) in the univariate analysis.

**TABLE 3:** Factors associated with common mental disorders among people living with HIV.

Characteristics	CMD							
	No	Yes	OR	95% CI	*p*	aOR	95% CI	*p*
**Age (years)**								
18–30	29	33	1.00	-	-	-	-	-
31–40	60	52	0.76	0.41–1.42	0.391	-	-	-
41–50	75	45	0.53	0.28–0.98	0.043	-	-	-
> 50	76	30	0.35	0.18–0.67	0.002	-	-	-
**Gender**								
Male	76	75	1.00	-	-	1.00	-	-
Female	164	85	0.53	0.35–0.79	0.002	0.61	0.37–0.99	0.025
**Viral load (copies/mL)**								
≤ 50	161	91	1.00	1.00	-	-	-	-
> 50	64	45	1.24	0.79–1.97	0.352	-	-	-
≤ 200	197	103	1.00	-	-	-	-	-
> 200	28	33	2.17	1.26–3.72	0.005	-	-	-
**Chronic diagnosis**								
No	144	112	1.00	-	-	1.00	1.00	-
Yes	96	48	0.64	0.42–0.98	0.042	3.16	1.07–9.29	0.035
**Adherence (previous 1 month)**								
No missed dose	176	84	1.00	-	-	1.00	1.00	-
1–2 missed doses	43	46	2.24	1.37–3.66	0.001	2.62	1.49–4.60	0.001
> 2 missed doses	18	25	2.91	1.51–5.63	0.002	2.44	1.15–3.08	0.015
**Chronic pain**								
No	189	117	1.00	1.00	-	-	-	-
Yes	51	40	1.27	0.79–2.03	0.328	-	-	-
**Psychosocial stressors**								
No	181	118	1.00	-	-	-	-	-
Yes	59	42	1.09	0.69–1.72	0.707	-	-	-
**Employment status**								
Regular	110	70	1.00	-	-	-	-	-
Non-regular	129	88	1.07	0.72–1.61	0.736	-	-	-
**Screened for CMD (last 2 clinic visits)**								
No	138	55	1.00	1.00	-	1.00	1.00	-
Yes	99	99	2.51	1.65–3.81	0.000	2.95	1.79–4.88	< 0.001
**Clinic attendance (last 6 months)**								
Over 80%	151	81	1.00	1.00	-	-	-	-
Less than 80%	85	71	1.58	1.02–2.36	0.036	-	-	-
**Presence of social support**								
Never	6	9	1.00	1.00	-	-	-	-
Sometimes	39	34	0.58	0.19–1.80	0.347	-	-	-
Always	191	113	0.39	0.14–1.14	0.085	-	-	-

CMD, common mental disorder; OR, odds ratio; aOR, adjusted odds ratio; CI, confidence interval.

We also assessed for the proportion of our cohort that was screened by healthcare practitioners for CMDs during their most recent two clinic visits. We assessed if patients had been screened for CMDs based on recall and on clinic notes. Sixteen per cent of our cohort was screened for depression, 14% for GAD and 40% for SUDs, based on participant recall. When clinic notes were reviewed, only 1% of patients screened for CMDs had the screening documented in their clinic files.

### Factors associated with depression in people living with HIV

The presence of chronic pain and viral load above 200 copies/mL was associated with screening positive for depression, in both the univariate and multivariate analyses (Table 4). However, screening patients for depression during clinical assessment was associated with significantly lower risk of screening positive for depression only in the univariate analysis (OR = 0.18; 95% CI: 1.42–4.36; *p* = 0.001).

**TABLE 4:** Factors associated with depression among people living with HIV.

Characteristics	Depression							
	No	Yes	OR	95% CI	*p*	aOR	95% CI	*p*
**Age (years)**								
18–30	47	15	1.00	-	-	-	-	-
31–40	87	26	0.94	0.45–1.94	0.859	-	-	-
41–50	109	12	0.34	0.15–0.79	0.012	-	-	-
> 50	93	14	0.47	0.21–1.06	0.068	-	-	-
**Sex**								
Male	137	14	1.00	-	-	1.00	-	-
Female	199	53	2.61	1.39–4.88	0.003	2.30	1.08–4.88	0.030
**Viral load (copies/mL)**								
≤ 50	219	35	1.00	-	-	-	-	-
> 50	87	23	1.65	0.92–2.96	0.090	-	-	-
≤ 200	258	40	1.00	1.00	-	1.00	-	-
> 200	48	18	2.42	1.28–4.57	0.006	2.49	1.16–5.35	0.019
**Chronic diagnosis**								
No	215	43	1.00	1.00	-	-	-	-
Yes	121	24	0.99	0.57–1.71	0.976	-	-	-
**Adherence (previous 1 month)**								
No missed dose	227	36	1.00	1.00	-	1.00	-	-
1–2 missed doses	67	22	2.07	1.14–3.76	0.016	2.77	1.36–5.63	0.005
> 2 missed doses	35	8	1.44	0.62–3.35	0.396	1.11	0.39–3.17	0.841
**Chronic pain (previous 1 month)**								
No	267	42	1.00	1.00	-	1.00	-	-
Yes	66	25	2.41	1.37–4.23	0.002	2.49	1.27–4.91	0.008
**Psychosocial stress**								
No	258	43	1.00	1.00	-	1.00	1.00	-
Yes	78	24	1.85	1.05–3.23	0.032	-	-	-
**Employment status**								
Regular source of income	161	21	1.00	1.00	-	1.00	1.00	-
None or irregular source of income	173	45	1.99	1.14–3.49	0.016	2.52	1.26–5.04	0.009
**Screened for CMD**								
No	174	21	1.00	1.00	-	1.00	-	-
Yes	153	46	0.18	1.42–4.36	0.001	3.85	1.94–7.66	0.000
**Clinic attendance (last 6 months)**								
< 80%	128	29	1.25	0.73–2.13	0.142	-	-	-
> 80%	198	36	1.00	1.00	-	-	-	-
**Presence of social support**								
Never	9	6	1.00	1.00	-	-	-	-
Sometimes	53	20	1.63	0.68–3.93	0.276	-	-	-
Always	268	39	1.71	0.76–3.85	0.198	-	-	-

CMD, common mental disorder; OR, odds ratio; aOR, adjusted odds ratio; CI, confidence interval.

### Factors associated with generalised anxiety disorder in people living with HIV

Being female (OR = 2.80; 95% CI: 1.43–5.45; *p* = 0.003) and having a viral load above 200 copies/mL (OR = 3.61; 95% CI: 0.91–6.86; *p* = 0.000) were significantly associated with GAD in our univariate analysis (Table 5).

**TABLE 5:** Factors associated with generalised anxiety disorder among people living with HIV.

Characteristics	GAD							
	No	Yes	OR	95% CI	*p*	aOR	95% CI	*p*
**Age (years)**								
18–30	43	19	1.00	-	-			
31–40	95	18	0.43	0.20–0.90	0.024	-	-	-
41–50	106	15	0.32	0.15–0.69	0.003	-	-	-
> 50	98	9	0.21	0.09–0.50	0.000	-	-	-
**Gender**								
Male	139	12	1.00	1.00	-	1.00		
Female	203	49	2.80	1.43–5.45	0.003	4.56	1.87–11.13	0.001
**Viral load (copies/mL)**								
≤ 50	225	29	1.00	1.00	-	-	-	-
> 50	87	23	2.05	1.13–3.74	0.019	-	-	-
≤ 200	266	32	1.00	1.00	-	1.00	1.00	-
> 200	46	20	3.61	1.91–6.86	0.000	4.54	2.07–9.97	0.000
**Chronic diagnosis**								
No	214	44	1.00	1.00	-	1.00	1.00	-
Yes	128	17	0.65	0.35–1.17	0.154	3.67	1.12–12.09	0.041
**Adherence (previous 1 month)**								
No missed dose	235	28	1.00	1.00	-	1.00	1.00	-
1–2 missed doses	70	19	2.27	1.20–4.32	0.039	2.57	1.18–5.60	0.043
> 2 missed doses	31	12	3.25	1.50–7.04	0.002	3.40	1.25–9.24	0.021
**Chronic pain (previous 1 month)**								
No	271	38	1.00	1.00	-	1.00	1.00	-
Yes	68	23	2.41	1.35–4.32	0.003	3.20	1.57–6.52	0.001
**Psychosocial stressors**								
No	271	38	1.00	1.00	-	-	-	-
Yes	81	21	1.69	0.94–3.03	0.078	-	-	-
**Employment status**								
Regular source of income	161	21	1.00	1.00	-	-	-	-
Non-regular source of income	178	48	1.72	0.97–3.04	0.061	-	-	-
**Screened for CMD (last 2 clinic visits)**								
No	171	24	1.00	1.00	-	-	-	-
Yes	168	31	1.31	0.74–2.33	0.035	-	-	-
**Clinic attendance (last 6 months)**								
> 80%	200	34	1.00	1.00	-	-	-	-
< 80%	134	23	1.01	0.57–1.79	0.974	-	-	-
**Presence of social support**								
Never	10	5	1.00	1.00	-	-	-	-
Sometimes	57	16	0.56	0.17–1.88	0.349	-	-	-
Always	268	39	0.29	0.09–0.90	0.032	-	-	-

CMD, common mental disorders; GAD, generalised anxiety disorder; OR, odds ratio; aOR, adjusted odds ratio; CI, confidence interval.

This association also persisted in the multivariate analysis. Similarly, those who screened positive for GAD were four times more likely to have an unsuppressed viral load, and were more likely to have suboptimal adherence when compared with those who did not screen positive for GAD (Table 5).

### Factors associated with substance use disorder in people living with HIV

There was no significant association in the univariate analysis between SUDs and the presence of social support or regular employment (Table 6). Females were 80% less likely to have SUDs (*p* = 0.000) when compared with males, in both the univariate and multivariate analyses. Further analysis revealed that males were 11 times more likely to have severe SUDs. Similarly, there was association between SUDs and having a clinic attendance less than 80% (OR = 1.69; 95% CI: 1.13–2.55; *p* = 0.011) (Table 6).

**TABLE 6:** Factors associated with substance use disorders among people living with HIV.

Characteristics	SUD							
	No	Yes	OR	95% CI	*p*	aOR	95% CI	*p*
**Age (years)**								
18 – 30	24	38	1.00	-	-	1.00	1.00	-
31 – 40	50	63	0.80	0.42–1.50	0.478	0.59	0.27–1.30	0.193
41 – 50	62	59	0.60	0.32–1.12	0.109	0.43	0.20–0.96	0.040
> 50	68	39	0.36	0.19–0.69	0.002	0.22	0.10–0.52	0.000
**Gender**								
Male	41	110	1.00	1.00	-	1.00	1.00	-
Female	163	89	0.20	0.13–0.32	0.000	0.19	0.11–0.32	0.000
**Viral load (copies/mL)**								
≤ 50	129	125	1.00	-	-	1.00	1.00	-
> 50	64	46	0.74	0.47–1.17	0.195	0.54	0.31–0.94	0.029
≤ 200	161	137	1.00	-	-	-	-	-
> 200	32	34	1.25	0.73–2.13	0.415	-	-	-
**Chronic diagnosis**								
No	124	134	1.00	-	-	-	-	-
Yes	80	65	0.75	0.45–1.13	0.171	-	-	-
**Adherence (previous 1 month)**								
No missed dose	148	115	1.00	-	-	1.00	1.00	-
1 – 2 missed doses	33	56	2.18	1.33–3.58	0.001	2.15	1.18–3.91	0.012
> 2 missed doses	21	22	1.20	0.89–1.71	0.364	0.81	0.36–1.85	0.618
**Chronic pain (previous 1 month)**								
No	156	153	1.00	-	-	-	-	-
Yes	48	43	0.91	0.57–1.46	0.704	-	-	-
**Psychosocial stressors**								
No	151	150	1.00	-	-	-	-	-
Yes	53	49	0.93	0.59–1.45	0.754	-	-	-
**Employment status**								
Regular source of income	88	94	1.00	1.00	-	-	-	-
Non-regular source of income	116	102	0.82	0.56–1.22	0.333	-	-	-
**Screened CMD**								
No	120	75	1.00	-	-	1.00	1.00	-
Yes	80	119	2.38	1.59–3.57	0.000	2.26	1.36–3.74	0.002
**Clinic attendance in last 6 months**								
≥ 80%	174	44	1.00	-	-	1.00	1.00	-
< 80%	151	22	1.69	1.13–2.55	0.011	1.688	1.02–2.79	0.041
**Presence of social support**								
Never	4	11	1.00	-	-	-	-	-
Sometimes	36	37	0.37	0.11–1.28	0.118	-	-	-
Always	161	146	0.33	0.10–1.06	0.062	-	-	-

CMD, common mental disorders; SUD, substance use disorder; OR, odds ratio; aOR, adjusted odds ratio; CI, confidence interval.

## Discussion

### Prevalence of common mental disorders and associated factors

We assessed the prevalence of CMDs and their associated factors in PLHIV in a South African health district. We found a high CMD prevalence of 40.0%, which was significantly associated with being male, viral non-suppression, younger age and having poor adherence. This prevalence was comparable with a study conducted in Ethiopia, where the CMD prevalence was 32.7%.^25^ It was, however, higher than in a study conducted in Zimbabwe, where the prevalence of CMDs was 18.0%.^26^ The lower prevalence in the Zimbabwean study may have been because of the higher sample size, which may underestimate the prevalence of common mental disorders. Another reason may be that different screening instruments were used in both studies. These instruments have different specificities, sensitivities and predictive values, for the detection of CMDs in PLHIV.

Also, since previous studies have reported changes in important risk factors for CMDs such as the number of PLHIV disclosing their HIV status to their families and sexual partners, HIV-associated stigma and treatment options, one may have expected a change in the prevalence of CMDs. For example, only 30.0% of women disclosed their HIV status to their sexual partners in 2015, increasing to 58.0% and 81.8% in 2018 and 2021, respectively.^27,28,29^ This was, however, not the case as the prevalence of CMDs in PLHIV remains high according to the findings of this index study. This may be explained by the fact that the coronavirus disease 2019 (COVID-19) pandemic coincided with our study period. This pandemic resulted in increased mortality, social isolation and loss of income, which are important risk factors for CMDs.^30,31^ These CMDs have been associated with inadequate ART adherence in primary and tertiary healthcare facilities in Mozambique, which was in agreement with our study.^32^ This poor adherence was associated with failure of HIV suppression. Also in agreement with our study were findings that the younger groups had higher incidence of CMD, while the level of formal education and employment status were not predictors of CMD.^33^

With regard to the prevalence of depression and associated factors, women were more than two times more likely to be depressed than men in our cohort. This was consistent with most studies reviewed.^34,35^ We also showed that screening patients for depression was associated with lower risk of screening positive for depression. This strengthens previous recommendation to screen PLHIV for depression and refer for management, when the screening is positive.^16^ Similarly, the presence of unsuppressed viral load, psychosocial stressors and poor adherence were associated with depression in our study and other studies reviewed.^35^ However, in an American study conducted by Joseph et al.,^36^ chronic pain was not associated with depression, unlike what was found in our index study where those with chronic pain were more than two times more likely to be depressed. The observed differences may be attributable to methodological differences such as the use of the PHQ-8 to assess for depression and the use of the Brief Pain Inventory for pain assessment, in the American study.^37^ The methodological variance may also explain differences found in several studies assessing the prevalence of depression in PLHIV. In our index study, only 16.6% screened positive for depression. This was much lower than in studies in the United States of America (USA) where 36.0% of PLHIV screened positive for depression.^38^ The University of Michigan Composite International Diagnostic Interview (UM-CIDI) and the DSM III were used in the American study. Another reason may be that lower cut-offs were used in this study which also included patients with mild depressive symptoms. A sub-Saharan African study including 13 studies from 7 countries, however, reported a pooled prevalence of 13.9% for major depressive disorder. This was quite similar to our index study.^39^

### Prevalence of generalised anxiety disorder and associated factors

Similarly, only 15.1% of our cohort screened positive for GAD in our study. This was comparable with studies conducted in Zambia and Brazil, where the prevalence of GAD was 13.3% and 14.0%, respectively.^40,41^ However, the prevalence of GAD was much higher in studies in the USA (31%) and Ethiopia (22%).^42,43^ A hospital setting was used in the Ethiopian study and the Beck Anxiety Inventory scale was used to assess for GAD. While the American study used the GAD-7, it adopted a higher cut-off score (10 versus 8 in our study) and used a higher sample size recruited over 4 years; their calculated prevalence was more than two-fold higher than our study. This difference may be attributable to sociocultural differences between study sites and the use of convenience sampling in the American study, which may bias their study.^37^

Regarding factors associated with GAD in PLHIV, lacking social support, being female and having perceived stigma, were all associated with symptoms of GAD.^42^ In a Brazilian study, having chronic pain or being female was also associated with a higher incidence of GAD.^36,41^ These findings were consistent with our study, where being female and chronic pain were associated with increased prevalence of GAD in our multivariate analysis. We also found that poor adherence and unsuppressed viral load were associated with increased symptoms of GAD. There was no association between adherence or viral load and GAD symptoms in the Brazilian study.^41^ This association was, however, made in the American and Malaysian studies respectively.^44,45^ The Malaysian study also found association between GAD and substance use.

### Prevalence of substance-use disorders and associated factors

Among our cohort, 24.1% screened positive for substance-use disorders. Most of these (48%) were mild SUDs, while 26% each had moderate and severe SUD. This was similar to a study carried out in Cape Town which used the Drug Use Disorder Identification Test (DUDIT) to assess for ‘problematic drug use’.^38,46^ In this Cape Town study, 20% of participants screened positive for SUDs, but the authors did not determine the severity of the SUDs in their sample. Regarding the types of substances used, we found that the most common substances used by our cohort were alcohol (42.9%) and tobacco (24.3%). Only 3.7% and 0.5% reported using cannabis and opioid, respectively.

Alcohol was also the most common substance used among PLHIV in Nigeria, followed by oral sedatives (21.0%), cannabis (3.6%) and cocaine, inhalants and solvents (0.26%).^47^ Alcohol followed by tobacco were also the most frequent substance used in Mthatha, South Africa.^48^ In our study, however, among those with severe SUD, the most common substance used was tobacco followed by alcohol, then cannabis.

Concerning factors associated with SUDs, participants who had less than 80% clinic attendance, had previously defaulted ART or unsuppressed viral load, were more likely to screen positive for SUDs in our study. Female participants were 80% less likely than males to screen positive for SUDs, in both our univariate and multivariate analyses. Also, males were 11 times more likely to have severe SUDs compared to females. Kader et al.,^46^ like in our study, reported that males were more likely to be hazardous substance users. This is consistent with SUDs in the general population.^49^ They also found that participants with SUDs were more likely to be unemployed, and poorly adherent. These have negative impact on ART outcomes. The US National Institute of Health (NIH) also observed that ‘substance use disorders (SUDs) are prevalent among PLHIV and contribute to poor health outcomes; therefore, screening for SUDs should be a routine part of clinical care’.^38,50^

### Healthcare worker adherence to recommendation to screen for common mental disorders

Only 16% of participants were screened for depression, 14% for GAD and 40% for SUDs based on participant recall. A review of clinical notes revealed that only 1% of those screened for CMDs had the screening documented in their clinical records.^37^ These figures were quite small considering that the SA NDoH recommends that all patients living with HIV be screened for CMDs before and during ART.^16^ This also reveals that healthcare workers do not always document when they screen patients for CMDs. A doctoral study, however, reported that only 10% of their cohort were screened for depression at baseline. This improved to 100% after a structured training, and this was associated with improvement in ART outcomes.^51^ Therefore, an educational intervention to teach healthcare workers to screen for CMDs may improve ART outcomes.

We further evaluated for association between those who were screened by healthcare practitioner for CMDs and the presence of a CMD. Unexpectedly, screening for CMDs in our study was not associated with reduction in the prevalence of CMDs. It instead appeared to be associated with higher prevalence of CMDs. This may be because patients who screened positive did not always receive the required treatment after being identified by screening tools.^52^ We, however, did not quantify who were referred for treatment after screening positive using our screening tools.

## Limitations of study

Some limitations were noted with our study. Some aspects of our questionnaire relied on participant recall, and it is known that patients with CMDs, especially depression, may have impaired ability to recall. The time frame for recall was reduced to the last two clinic visits to reduce the probability of poor recall. Also, the instruments used for the study were screening, and not diagnostic tools. This may have also influenced the study findings. It must, however, be stated that the chosen screening tools have been validated locally and found to have good psychometric properties, and the DSM-5 criteria, which was used to screen for SUDs, is the gold standard for the diagnosis of SUDs. Another limitation was that of incomplete variable data. However, apart from the number of participants having viral load data, the total number of all other variables did not affect the power of the study. Finally, another limitation of our study was by not assessing the proportion of those who screened positive for a CMD and were referred for further assessment and management. This may guide future research.

Irrespective of the above limitations, this was a study involving patients managed across multiple primary health care facilities.^37^

## Conclusion

The findings of our study suggest a high prevalence of CMDs in PLHIV. It also found that those without CMDs have better ART outcomes than those with CMDs. These outcomes include viral suppression, adherence to treatment and clinic attendance. We have also shown that being female, experiencing HIV-associated stigma, experiencing chronic pain, lacking social support or having other chronic diagnosis, are associated with CMDs. Furthermore, the adherence to SA NDoH recommendation to screen for CMDs in PLHIV is very low. Therefore, systems need to be developed to motivate healthcare practitioners to screen for and manage CMDs in PLHIV. Further research may be necessary to guide how this should be implemented.
